# Development of Models for Predicting the Predominant Taste and Odor Compounds in Taihu Lake, China

**DOI:** 10.1371/journal.pone.0051976

**Published:** 2012-12-19

**Authors:** Min Qi, Jun Chen, Xiaoxue Sun, Xuwei Deng, Yuan Niu, Ping Xie

**Affiliations:** 1 Donghu Experimental Station of Lake Ecosystems, State Key Laboratory of Freshwater Ecology and Biotechnology of China, Institute of Hydrobiology, Chinese Academy of Sciences, Wuhan, Hubei, People’s Republic of China; 2 Fisheries College of Huazhong Agricultural University, Wuhan, Hubei, People’s Republic of China; University of Vigo, Spain

## Abstract

Taste and odor (T&O) problems, which have adversely affected the quality of water supplied to millions of residents, have repeatedly occurred in Taihu Lake (e.g., a serious odor accident occurred in 2007). Because these accidents are difficult for water resource managers to forecast in a timely manner, there is an urgent need to develop optimum models to predict these T&O problems. For this purpose, various biotic and abiotic environmental parameters were monitored monthly for one year at 30 sites across Taihu Lake. This is the first investigation of this huge lake to sample T&O compounds at the whole-lake level. Certain phytoplankton taxa were important variables in the models; for instance, the concentrations of the particle-bound 2-methylisoborneol (p-MIB) were correlated with the presence of *Oscillatoria*, whereas those of the p-β-cyclocitral and p-β-ionone were correlated with *Microcystis* levels. Abiotic factors such as nitrogen (TN, TDN, NO_3_-N, and NO_2_-N), pH, DO, COND, COD and Chl-a also contributed significantly to the T&O predictive models. The dissolved (d) T&O compounds were related to both the algal biomass and to certain abiotic environmental factors, whereas the particle-bound (p) T&O compounds were more strongly related to the algal presence. We also tested the validity of these models using an independent data set that was previously collected from Taihu Lake in 2008. In comparing the concentrations of the T&O compounds observed in 2008 with those concentrations predicted from our models, we found that most of the predicted data points fell within the 90% confidence intervals of the observed values. This result supported the validity of these models in the studied system. These models, basing on easily collected environmental data, will be of practical value to the water resource managers of Taihu Lake for evaluating the probability of T&O accidents.

## Introduction

Algal blooms have occurred frequently and intensively around the world in recent decades [1]. During their growth and decay, these algae can produce unwanted metabolites, such as biotoxins and/or T&O compounds [2]–[5]. Currently, the T&O compounds are causing global concerns because they influence the use, aesthetic and economic values of the waters. Especially, massive occurrences of T&O compounds in lakes and reservoirs frequently lead to odor pollution of both drinking water and aquatic products [2], [6]–[9], greatly prompting consumer complaints [10], [11] and economic losses [12]. Thus, considerable economic and social benefit would accrue if the development of unwanted T&O contamination could be predicted before full outbreaks occur. Fortunately, a few studies suggest that it is feasible to predict the occurrence of off-flavors using certain closely related environmental factors that can be measured by simple and low-cost methods [9], [13], [14]. Clearly, it is of practical value to use models with easily detectable parameters to predict the occurrence of T&O events a few days before they occur, which is undoubtedly important for the implementation of appropriate and timely measures by departments responsible for the management of water quality.

The production and occurrence of objectionable T&O compounds are known to be correlated with various biological and environmental factors, such as phytoplankton [15]–[18], nutrient concentrations and their ratios [13], [19], water temperature, pH and dissolved oxygen [16], [20], [21]. Nevertheless, only a few studies have reported models constructed to identify the key factors determining the T&O events. For example, Dzialowski et al. [13] constructed models for dissolved geosmin (d-GEO) in the drinking water reservoirs of Kansas (USA) using several abiotic factors and four cyanobacterial genera. Sugiura et al. [22] developed predictive models for dissolved 2-methylisoborneol (d-MIB) and d-GEO using data on abiotic environmental parameters and 25 genera of phytoplankton collected from Lake Kasumigaura (Japan). However, there are still many unknowns concerning the development of predictive models for the T&O compounds. First, most previous investigations concentrated on the dissolved compounds [9], [14], with little attention to those in the particulate form, which could be massively released into the water when algal cells are damaged [18], [23] and cause secondary pollution. Second, previous models were developed without distinguishing the different algal growing seasons [14], [24], which are important to distinguish because the blooming and nonblooming seasons in many eutrophic waters like Taihu Lake differ markedly in both the composition and the abundance of the phytoplankton community. Third, most studies have examined only the well-known earthy-musty algal metabolites, GEO and MIB, and just a few have studied other algal metabolites or derivatives, such as β-ionone [25], [26], β-cyclocitral [25], [27], 2-isobutyl-3-methoxypyrazine (IBMP) [28], [29], 2-isopropyl-3-methoxypyrazine (IPMP) [29], [30], dimethyl trisulfide and related alkyl sulfide compounds [31].

The present study was conducted in Taihu Lake, which has regular cyanobacterial blooms [32], [33] and experienced serious odor-caused drinking water pollution in the summer of 2007 when a dense cyanobacterial bloom occurred [34]. The seasonal and spatial dynamics of the T&O compounds, diverse physicochemical parameters and the biomass of various phytoplankton groups were monitored monthly during June 2009 to May 2010. The purpose of the present research was to utilize this massive collection of biotic and abiotic data gathered in both the blooming and nonblooming seasons to construct a series of models to predict the quantities of the predominant dissolved and particle-bound T&O compounds likely to develop in the lake. We attempted to identify the key environmental factors responsible for the development of the T&O compounds and to optimize the predictive models so that water resource managers can take appropriate and timely measures to prevent unwanted T&O outbreaks.

## Results

### Water Quality Conditions

**Figure 1:**
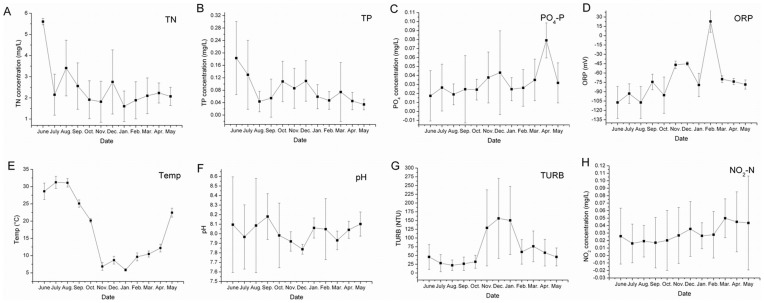
Annual variation of water physicochemical variables from June 2009 to May 2010. (Data represent monthly averages from thirty samples).

**Figure 2:**
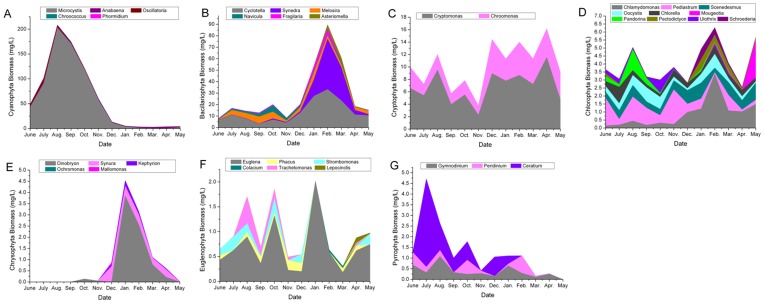
Phytoplankton community composition in Taihu Lake from June 2009 to May 2010. (Data represent only the main genera and are the monthly total biomass from thirty samples).

The annual variation in the mean monthly value of several basic physicochemical variables is shown in Figure 1. The pH, PO_4_-P, and NO_2_-N values showed relatively small ranges compared with the other parameters, with monthly averages from 7.84 to 8.18, 0.02 to 0.08 mg/L, and 0.02 to 0.05 mg/L, respectively. The maximum average temperature was 23.84°C in the blooming season and 11.53°C in the nonblooming season. The monthly averages of the TURB ranged from 22.09 NTU in July to 156.11 NTU in December. The concentrations of the TN and the TP were extremely variable at several sampling sites and during some months (Fig. 1); for example, the TN in August and September and the TP in June. Most of the concentrations ranged from 0.34 ∼ 3.54 mg/L for the TN and 0.04 ∼ 0.46 mg/L for the TP. The Cyanophyta composed the greatest proportion of the total phytoplankton biomass (55.9%), followed by the Bacillariophyta (27.9%). Cyanophyta had huge amounts of biomass in the blooming season, with *Microcystis* being the dominant genus. The biomass of the Bacillariophyta and Chrysophyta was greater in the nonblooming than in the blooming season. Chlorophyta owned varieties of taxa throughout the study period (Fig. 2).

### Data Analysis of the T&O Compounds

**Figure 3:**
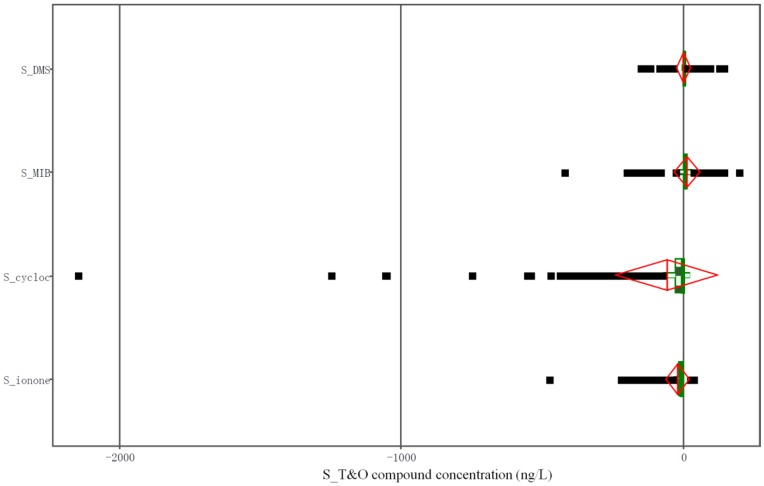
Boxplots for four S_T&O compounds. S_DMS = (d-DMS) - (p-DMS), S_MIB = (d-MIB) - (p-MIB), S_cycloc = (d-β-cyclocitral) - (p-β-cyclocitral), S_ionone = (d-β-ionone) - (p-β-ionone), S_T&O compound = (dissolved T&O compound) - (particulate T&O compound).

The boxplots (Fig. 3), coupled with the variance analyses, showed that the concentrations of the d-β-cyclocitral and the d-β-ionone were significantly lower than those of the p-β-cyclocitral and the p-β-ionone, respectively. In contrast, there was more MIB in the dissolved than in the particulate fraction. For DMS, no significant difference was found between the two fractions. Seasonally, the concentration of the d-β-cyclocitral was lower in the winter than in the late spring and early summer (the highest value was 49 ng/L). For the p-β-cyclocitral, the concentration was lower in the winter and spring than in the summer and autumn (the peak value was 2155 ng/L). The p-β-ionone showed a trend similar to that of the p-β-cyclocitral, whereas the d-MIB concentration was higher in the spring and autumn than in the summer and winter. The highest p-MIB concentration occurred in July. The monthly average d-DMS concentration was higher in the summer than in the other seasons, with a peak value of 22 ng/L in August. The maximum d-DMS value recorded was 143 ng/L.

### Model Development

#### Models for d-DMS

The d-DMS level was positively related to the levels of the TDN and the *Planktolyngbya* (Cyanophyta) and the *Cocconeis* (Bacillariophyta) algal genera, and negatively related to the NO_2_-N level during the blooming season (Table 1, Eq. (1)). In this season, *Planktolyngbya* level was strongly associated with the d-DMS level and showed the greatest relative weight of 0.6832. The TDN and the NO_2_-N were more weakly associated with the d-DMS, with relative weights of 0.0344 and 0.1065, respectively. During the nonblooming season, the d-DMS was negatively related to the TN, but positively related to the TDN, NO_3_-N and ORP (Table 1, Eq. (2)). The NO_3_-N was strongly associated with the d-DMS in this season and was the variable with the greatest relative weight (0.3176), followed by the TDN and TN (0.2676 and 0.2338, respectively). The environmental factors in the models explained 83.50 and 71.28% (R^2^) of the variations in the d-DMS levels during the blooming and nonblooming seasons, respectively (Table 1, Eqs. (1) and (2)).

**Table 1:** Statistical equations for the predominant T&O compounds in Taihu Lake models.

Season	Models	R^2^	Adj R^2^	Sign
B	d-DMS = 3.8245 TDN –36.5282 NO_2_-N +1256.41 *Planktolyngbya* +100.694 *Cocconeis*	0.8350	0.8302	Eq. (1)
N	d-DMS = –1.9913 TN +6.1900 TDN +28.0145 NO_3_-N +0.0120 ORP	0.7128	0.7070	Eq. (2)
B	d-MIB = –4.3869 NO_3_-N +0.216 COND +391.325 *Cosmarium* +745.074 *Phormidium*	0.7913	0.7892	Eq. (3)
N	d-MIB = 0.1960 COND –0.3861 TDS –9.5015 pH +8.1671 DO +5.1561 Bacillariophyta	0.7096	0.7003	Eq. (4)
B	p-MIB = –0.0020 COND –0.0798 COD +108.050 *Oscillatoria*	0.7728	0.7679	Eq. (5)
N	p-MIB = 26.6792 *Oscillatoria* +4.2806 *Pectodictyon* +0.2150 *Synedra*	0.8943	0.8927	Eq. (6)
B	d-β-cyclocitral = 0.5512 TN +0.0550 Chl-a –1.3141 *Pediastrum*	0.7549	0.7493	Eq. (7)
N	d-β-cyclocitral = 0.0354 DO –0.0505 *Cryptomonas* +603.952 *Ochromonas*	0.5322	0.5211	Eq. (8)
B	p-β-cyclocitral = 2.7228 TN +5.5838 Chl-a +4.8124 *Microcystis*	0.8554	0.8522	Eq. (9)
N	p-β-cyclocitral = 16.6857 *Microcystis* +48.7448 *Chlorella*	0.8942	0.8932	Eq. (10)
B	p-β-ionone = 195.500 NO_2_-N +1.1645 Chl-a +3.6887 *Microcystis*	0.8824	0.8799	Eq. (11)
N	p-β-ionone = 0.2642 DO +0.3474 COD +5.1278 *Microcystis*	0.8884	0.8861	Eq. (12)

All p-values <0.0001; B, Blooming season; N, Nonblooming season.

#### Models for d-MIB and p-MIB

The concentration of the d-MIB was negatively related to that of the NO_3_-N but positively related to the levels of the COND, *Cosmarium* (Chlorophyta) and *Phormidium* (Cyanophyta) during the blooming season (Table 1, Eq. (3)). For the d-MIB, *Cosmarium* showed the greatest relative weight (0.5863), followed by the NO_3_-N (0.0721). During the nonblooming season, the d-MIB concentration was mainly determined by a series of abiotic and biotic factors, which included the pH, the DO, and the Bacillariophyta (Table 1, Eq. (4)); however, the biotic factor (Bacillariophyta) was relatively more important (weight of 0.6713) than the abiotic factors. The parameters in these two models explained 79.13 and 70.96% of the variation in the d-MIB concentration in the blooming and nonblooming seasons, respectively.

During the blooming season, the p-MIB level was mainly related positively to that of *Oscillatoria* (Cyanophyta), but also negatively to the COND and COD levels, and these three variables accounted for 77.28% of the variation in the p-MIB (Table 1, Eq. (5)). For predicting the p-MIB concentration in the blooming season, the *Oscillatoria* level was the most important of the variables, with a relative weight of 0.8770. During the nonblooming season, the p-MIB was positively related to *Oscillatoria*, *Pectodictyon* (Chlorophyta) and *Synedra* (Bacillariophyta), with *Oscillatoria* having the greatest relative weight (0.4706). These biotic factors accounted for up to 89.43% of the variation in the p-MIB concentration (Table 1, Eq. (6)).

#### Models for d-β-cyclocitral and p-β-cyclocitral

Generally, the concentration of the d-β-cyclocitral was positively related to the TN and Chl-a levels, but negatively related to the *Pediastrum* (Chlorophyta) biomass, which was the most important predictive variable during the blooming season (Table 1, Eq. (7)). For predicting the d-β-cyclocitral concentration during this period, the TN level displayed a relative important weight of 0.1118. The d-β-cyclocitral concentration was positively related to the DO and the *Ochromonas* (Chrysophyta) levels, but negatively related to the *Cryptomonas* (Cryptophyta) level during the nonblooming season (Table 1, Eq. (8)). The *Ochromonas* level was the most useful (weight of 0.9010) for predicting the d-β-cyclocitral concentration in the nonblooming season. The model variables accounted for 75.49% (Eq. (7)) and 53.22% (Eq. (8)) of the variation in the d-β-cyclocitral concentration during the blooming and nonblooming seasons, respectively.

The p-β-cyclocitral level was related significantly and positively to that of *Microcystis* (Cyanophyta) throughout the year (Table 1, Eqs. (9) and (10)), and the *Microcystis* level was the best predictor of the p-β-cyclocitral concentration during the whole year. Additionally, the p-β-cyclocitral concentration was positively related to the TN and Chl-a levels during the blooming season and positively related to the *Chlorella* (Chlorophyta) biomass during the nonblooming season. The relative weight of the TN level for predicting the p-β-cyclocitral concentration was 0.1393 during the blooming season. These models accounted for 85.54 and 89.42% of the variation in the p-β-cyclocitral concentration in the blooming and nonblooming seasons, respectively (Table 1, Eqs. (9) and (10)).

#### Models for p-β-ionone

The p-β-ionone concentration was positively related to the NO_2_-N, the Chl-a, and *Microcystis* levels during the blooming season (Table 1, Eq. (11)), and related positively to the DO, the COD, and *Microcystis* levels in the nonblooming season (Table 1, Eq. (12)). The *Microcystis* level was the best predictor of the p-β-ionone concentration throughout the year. For predicting the p-β-ionone concentration during the blooming season, the NO_2_-N level showed a relative weight of 0.3402. These models accounted for 88.24 and 88.84% of the variation in the p-β-ionone concentration in the blooming and nonblooming seasons, respectively (Table 1, Eqs. (11) and (12)).

#### Diagnosis and test for the models

**Figure 4:**
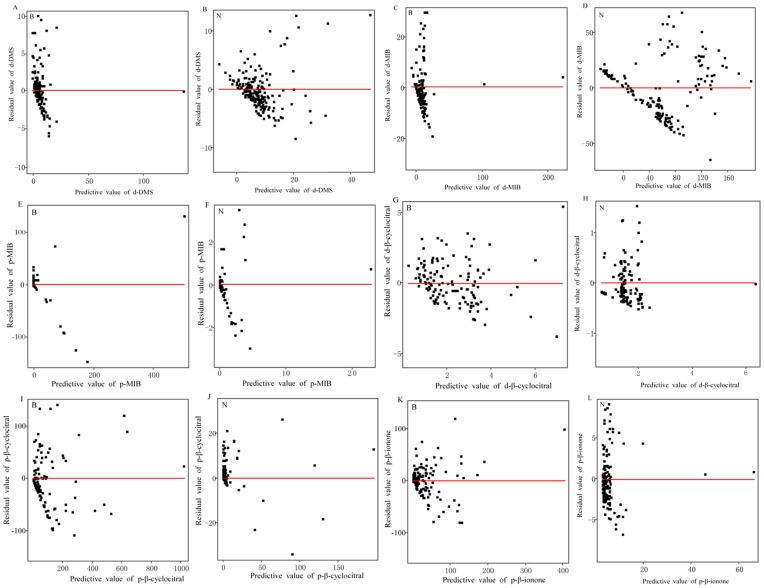
Residual scatter plots for T&O compound models. B = blooming season, N = nonblooming season.

In the scatter plots of the residuals (Fig. 4), a random dispersal of the data points around the zero line would indicate that the developed models can be considered appropriate. Fortunately, the data points in most of the plots in Fig. 4 were randomly distributed. Although some of the plots displayed scatter points slightly away from the zero line, they did not show a clear tendency to be curved (e.g., U-shaped and inverted U). Therefore, the scatter plots provided little evidence to question the appropriateness of the linear regression models, and the models for the T&O compounds were considered to be valid.

**Figure 5:**
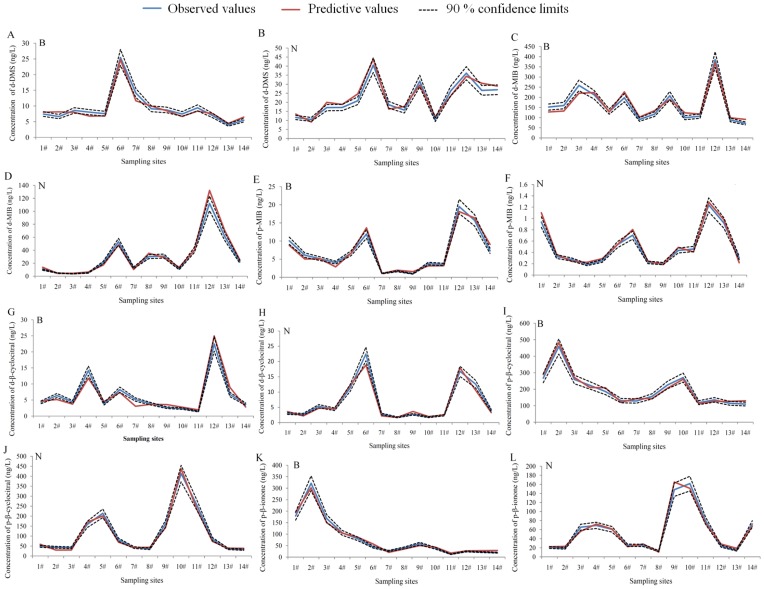
Test of the predictive models for predominant T&O compounds in Taihu Lake. B = blooming season, N = nonblooming season.

The test results with the independent data set collected from Taihu Lake in 2008 showed that most of the values predicted with the models fell within the 90% confidence intervals of the observed values (Fig. 5). For instance, 85.7 ∼ 92.9% of the predicted p-β-cyclocitral and p-β-ionone data points fell within the 90% confidence intervals of the observed values in both the blooming and nonblooming seasons. The timing and intensity of these odors were well predicted with a few deviations. Therefore, models developed here could predict well the occurrences and levels of the corresponding odors in Taihu Lake.

## Discussion

### The Importance of Developing Predictive Models for Both Dissolved and Particle-bound T&O Compounds

In the present study, the dissolved T&O compounds in the water column were closely related to both the algal biomass and the abiotic environmental factors, whereas the particle-bound T&O compounds were more strongly related to the algae. For some of the T&O compounds, such as β-cyclocitral, both fractions should react similarly and produce similar models because the dissolved fraction is released from the particulate fraction present in the algal cells [15], [35]. However, the dissolved odors in water are easily influenced by environmental processes, such as biodegradation [8], [36], photolysis [36], [37], volatilization [8] and sorption [8], [38]. In addition, the synthetic pathways of some odors might also vary between the two fractions. The dissolved forms might originate from compounds other than the corresponding cellular T&O compounds, e.g., the d-DMS. Dimethylsulfoniopropionate is produced intracellularly by many algae [39], and then it is transferred extracellularly [40] and subsequently transformed rapidly into DMS through physicochemical degradations [16]. It is likely that these might have greatly affected the correlations between the dissolved and particulate T&O fractions, consequently leading to our use of the different models for the two forms of the T&O compounds.

Furthermore, the dissolved T&O fraction could directly influence water quality, and the particle-bound fraction in the algal cells could become an important source of T&O problems when the cells are damaged or decomposed. Thus, it was necessary to establish a separate model for each form of the T&O compounds.

### Influences of Phytoplankton on the Predictive Models

Some of the T&O compounds (e.g., DMS, MIB, β-cyclocitral, and β-ionone) are often assumed to be produced by specific algae, bacteria, or fungi. Such organisms include *Oscillatoria* for the MIB in Eqs. (5) and (6), and *Microcystis* for the β-cyclocitral and β-ionone in Eqs. (9) ∼ (12). However, some algae that have not been reported to produce odors were included in the models; for example, Bacillariophyta in Eq. (4), *Pectodictyon* and *Synedra* in Eq. (6), and *Chlorella* in Eq. (10). These algae contributed greatly to the models, as demonstrated by their large relative weights: 0.6713 for the effect of the Bacillariophyta on the d-MIB in Eq. (4); 0.2742 and 0.2552 for the effect of the *Pectodictyon* and *Synedra*, respectively, on the p-MIB in Eq. (6); and 0.3376 for the effect of *Chlorella* on the p-β-cyclocitral in Eq. (10). Apparently, the growth and extinction of these algae, which are not considered to be producers of the T&O compounds, influenced the T&O producers or the physicochemical properties of the water and consequently, influenced the T&O compounds indirectly. Recent studies have also reported that some algae that were previously considered to be incapable of producing odors could be influenced by these off-flavors [41], [42]. For example, it has been reported that diatom as well as cyanobacteria could be used by *Streptomyces* from sediment as a C source to produce MIB [43], [44]. In the study by Sugiura et al. [22], the MIB levels were significantly correlated with the concentrations of the green algae and the diatoms in Lake Kasumigaura. It is known that *Microcystis* can produce the β-cyclocitral and β-ionone [2], [35], and Sommerburg et al. [45] demonstrated that the oxidative cleavage of β-carotene can generate both compounds. There is also speculation that phytoplankton species (such as *Chlorella* in Eq. (10)) that contain β-carotene have the potential to produce the β-cyclocitral and β-ionone [46]. Because β-carotene exists widely in all phytoplankton, the β-carotene contained in these algae (e.g. *Pediastrum* in Eq. (7), *Cryptomonas* in Eq. (8) and *Chlorella* in Eq. (10)) can likely be transformed into β-cyclocitral and β-ionone. In summary, the odor-producing algae played important roles in the dynamics of the T&O compounds in Taihu Lake, and other phytoplankton species might also have substantial influences on these compounds. Thus, algal species besides the odor-producing algae should also be considered when investigating the T&O compounds.

### Influences of Physicochemical Parameters on the Predictive Models

Similar to the results of many previous studies, the current results showed that abiotic factors are important for predicting the occurrence of the T&O compounds in Taihu Lake. Notably, most of the models developed in the present study included various nitrogen forms (e.g., Eqs. (1), (2) and (11)). A previous investigation by our laboratory also closely associated the production of the T&O compounds in Taihu Lake with the nitrogen levels [27]. The nitrogen could affect the concentrations of the T&O compounds directly or indirectly [16], [39]. Ye et al. demonsrated that the TN was the best predictor of the peak algal biomass from 1998 to 2008 in Taihu Lake by constructing a modified Monod model with the TN: TP ratio of approximately 35∶1 [47]. In their study period, the annual average concentration of the TP ranged from 0.014 ∼ 0.20 mg/L, which was similar to our average TP concentrations (0.03 ∼ 0.19 mg/L). It has also been reported that nitrogen limits the phytoplankton growth in Taihu Lake during the summer and fall months [48]. Thus, it is likely that nitrogen might indirectly affect the production and release of the T&O compounds by algae. Additionally, Yang et al. showed that the nitrogen form preferentially absorbed by the algae differed in the different sections of Taihu Lake [49]. Therefore, changes in the available nitrogen forms and their ratios may also influence the phytoplankton and the subsequent production of the T&O compounds. The great heterogeneity is also a characteristic of this huge lake. Regardless of the mechanisms involved, it should be stressed that the levels of the various forms of nitrogen were closely related to the levels of the T&O compounds in Taihu Lake.

### Model Test and Extension

The use of the independent data set to test the accuracy of the models showed that the occurrence and intensity of these T&O compounds were satisfactorily predicted, though there were several deviations. In a body of water as large and spatially heterogeneous as Taihu Lake, the environmental and climatic conditions may substantially vary both spatially and temporally. In addition, it is known that the volatility of the compounds producing the off-flavors causes their concentrations to vary easily in aquatic environments. The above phenomena could certainly result in discrepancies between the predicted and observed values. Nevertheless, the models developed in the present study accurately predicted the levels of a number of the T&O compounds in Taihu, which supplies water for drinking, industry and agriculture to millions of people. Therefore, these models, basing on easily collected environmental data, are of practical value to water resource managers for evaluating the probability of T&O accidents in Taihu Lake.

Previous researchers reported that the dynamics of odor compounds strongly depend on the local environmental conditions and vary from system to system [9], [13], [14]. Even for the same T&O compound, different models were generated in different reservoirs of the same region; thus, it is difficult to obtain a universal model applicable to all ecosystems [9], [13], [14]. It was not possible to test our models in other systems because of the lack of sufficient data, as we mentioned above. Nevertheless, models developed here have good utilities in such a huge lake based on the quite informative data in different algal growth seasons for different fractions of the T&O compounds. We believe that the models in the present study should provide insights into developing a general model applicable to a variety of lake systems, especially other shallow eutrophic lakes with *Microcystis* blooms.

### Conclusion

We developed T&O compound predictive models using massive data of various abiotic and biotic parameters from Taihu Lake. Most of these models achieved a good fit and proved adequate for mapping the practical dynamics of the T&O compounds. Different from previous odor models, we considered two algal growth seasons (blooming and nonblooming) and two fractions of the T&O compounds (dissolved and particle-bound). These attributes contributed to the accuracy and sensitivity of the models. The dissolved T&O compounds varied with the algal biomass and with a variety of abiotic factors, whereas the particle-bound T&O forms varied primarily with the algal biomass. The use of a previously collected independent data set to test the equations showed that the models could accurately predict the concentrations of the T&O compounds in Taihu Lake, supporting the utility of these models in the studied system. Because Taihu supplies water for drinking, industry and agriculture to millions of people and because the models only require the collection of certain basic environmental data, these models are of practical value to the water resource managers for predicting the probability of T&O accidents.

## Materials and Methods

### Materials and Reagent

The T&O standard compounds, including DMS, dimethyl trisulfide (DMTS), IBMP, IPMP, GEO, MIB, β-cyclocitral, and β-ionone, were obtained from Sigma-Aldrich (Milwaukee, WI, USA). Freshly mixed 1 mg/L standard solutions were prepared in methanol (Merck, Germany, HPLC grade) before daily use. The dilution series used to generate the standard curves were prepared by diluting the standard solutions with HPLC grade water. Sodium chloride (Sinopharm Chemical Reagent, China, AR) was dissolved in HPLC grade water to yield a solution with a concentration of 250 g/L. This solution was used to extract the particulate T&O compounds. Whatman glass fiber filters (GF/C, Whatman, Brentford, UK) were used to separate the dissolved and particulate T&O compounds in the lake water.

### Lake Sampling and Data Collection

The study was conducted in Taihu Lake, a freshwater shallow lake with an area of 2338 km^2^ in China. At our sampling sites, the average depth was 1.9 m, and the maximum depth was 2.6 m. The lake supplies the water needs of the region, supporting approximately 10 million residents [50]. In recent decades, Taihu Lake has experienced serious pollution and anthropogenic eutrophication [51], severely influencing the normal life of citizens. For example, a 2007 incident in Wuxi City involving malodorous drinking water seriously influenced the water usage of approximately two million citizens for nearly one week [34].

**Figure 6:**
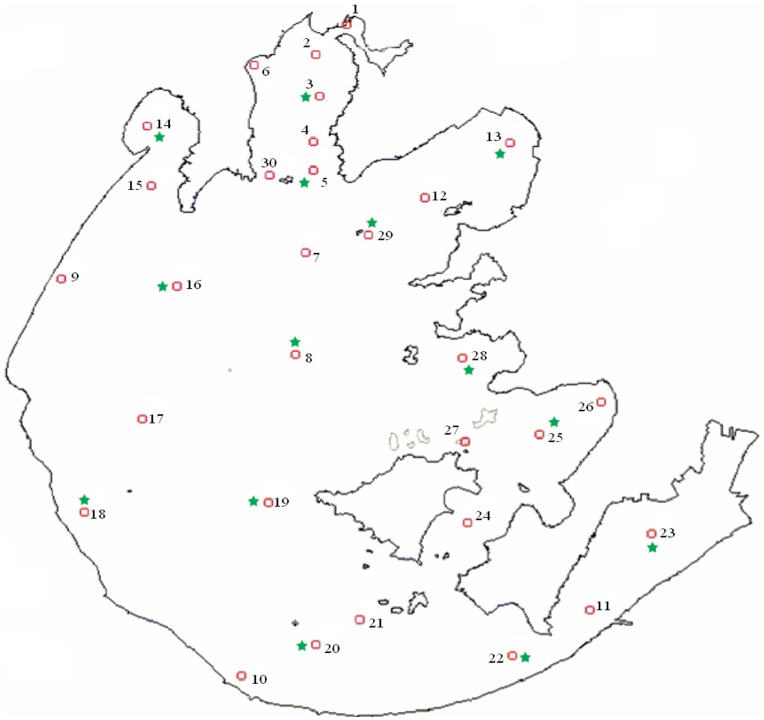
Sampling sites in Taihu Lake. The red circles represent the sampling sites in June 2009 to May 2010. The green stars represent the sampling sites in 2008.

The full lake survey was conducted from June 2009 to May 2010. Water samples were collected from 30 sampling sites (Fig. 6), which represented the distributions of the water-quality conditions and the T&O compound concentrations at the whole-lake level. Each water sample from each site was a mixture of two subsamples: one from 0.5 m below the surface and one from 0.5 m above the bottom. The study measured the concentrations of the T&O compounds and a variety of environmental physicochemical parameters, which included water temperature (Temp), water depth (Wd), transparency (SD), dissolved oxygen (DO), conductivity (COND), pH, turbidity (TURB), oxidation reduction potential (ORP), total dissolved solid (TDS) and chemical oxygen demand (COD), as well as hydrochemical indices. The hydrochemical indices, including total nitrogen (TN), total phosphorus (TP), total dissolved nitrogen (TDN), total dissolved phosphorus (TDP), nitrate nitrogen (NO_3_-N), nitrite nitrogen (NO_2_-N), ammonium (NH_4_-N), and orthophosphate (PO_4_-P), were analyzed using the standard colorimetric methods described by Strickland and Parsons [52]. The COD was determined through titration with sodium thiosulfate [53]. The concentration of chlorophyll-a (Chl-a) was determined spectrophotometrically [54]. The other abiotic factors were determined at the time of sampling. The analyses of the T&O compounds and the hydrochemical indices were completed within 24 h after sampling. For the phytoplankton analysis, one liter of the mixed water sample was immediately fixed with Lugol’s solution when sampling and then concentrated to 50 ml after sedimentation for 48 h. The taxonomic identification was conducted according to Utermoehl [55]. The algal biomass was estimated from cell numbers and cell sizes measurements. It was assumed that 1 mm^3^ of algal volume equals 1 mg of fresh-weight biomass. The *Microcystis* colonies were separated using an ultrasonic device before the biomass estimation. If there were large quantities of algae, the mixed water samples were fixed and counted directly without concentration.

Many techniques exit to extract the T&O compounds, such as SE [56], CLSA [57], SPME [58], [59] and purge-and-trap (P&T) [60]. Based on our previous studies [61], [62], the P&T extraction device coupled with GC-MS was chosen to determine the T&O compound concentrations in the present study. The dissolved T&O compounds in the filtrate of the water samples were directly determined using an Eclipse 4660 Purge-and-Trap Sample Concentrator and a 4551A autosampler (O.I. Analytical Company, USA) coupled with a gas chromatograph mass spectrometer (QP2010plus, Shimadzu Corporation, Japan) [61]. The oven temperature program was as follows: 50°C for 2 min, 10°C/min to 150°C and then 5°C/min to 220°C. The ion monitoring mode of the mass spectrometer was selected for better detection [61]. The ions selected for the T&O compounds are listed in Table 2. The filter residue containing the particulate T&O compounds was analyzed using the method described by Chen et al. [27].

**Table 2:** Concentration of T&O compounds in Taihu Lake.

Dissolved-form/Particle-form	Selected ions (m/z)	Range (ng/L)	Mean (ng/L)
DMS	62^a^, 47	0–143.2/0–154.3	7.8/5.5
DMTS	126^a^, 79, 111	0–13.3/0–28.7	0.5/1.5
IPMP	137^a^, 152, 124	0–6.8/0–31.0	0.3/0.3
IBMP	95^a^, 108, 135	0–2.0/0–25.3	0.3/0.5
MIB	124^a^, 94, 151	0–228.17/0–651.2	21.2/6.8
β-cyclocitral	137^a^, 152, 123	0–49.3/1.1–2155.0	3.0/61.1
GEO	112^a^, 125, 149	0–13.3/0–13.3	1.1/0.3
β-ionone	177^a^, 92, 135	0–81.3/0–223.1	1.3/20.6

a Quantitative ion.

### Model Development

Based on our previous studies [27], [61], DMS, DMTS, IPMP, IBMP, MIB, β-cyclocitral, GEO, and β-ionone were monitored. Variance analysis was conducted and boxplots were drawn for the main T&O compounds (d-DMS, d- and p-MIB, d- and p-β-cyclocitral, and p-β-ionone) (Fig. 3). The T&O compounds present at low concentrations (d- and p-GEO, d- and p-DMTS, d- and p-IPMP, d- and p-IBMP, p-DMS and d-β-ionone (Table 2)) were not considered in the following model development. Based on the phytoplankton biomass and the basic water-quality parameters in Taihu Lake, the whole year was divided into two periods: the blooming season (June to November) and the nonblooming season (December to May). Then, we constructed models for the dominant T&O compounds using multiple linear regression analyses, which are widely used in odor studies [22], [63]. The analyses were conducted using SAS/INSIGHT of SAS statistical software version 9.1 [64] on the biotic (phytoplankton) and abiotic (physicochemical) environmental parameters collected during the two seasons. Each model used the concentration of a T&O compound as the dependent variable (Y) and the other environmental parameters as the independent variables (X). Then, the parameters with the smallest F statistical value and an insignificant p-value were deleted using the backward elimination procedure. The elimination procedure was repeated until all the variables remaining in the model had probabilities below the significance level.

To determine the best-fit model, three primary factors were considered. The p-value (in the significance tests of the regression model) is the first limiting factor in selecting the best-fit model. Only statistically significant models with p-values less than α (significant level) were retained for further consideration. Second, the R-Sq (coefficient of determination) and the Adj R-Sq (the adjusted R-Sq, which considers the sample size (n) and the number of parameters (k) in the model) are indicators for evaluating the quality of models, as well as the III test table (a test of significance for the results of the hypothesis of each independent variable at zero; data not shown) and the parameter estimates table (a test of significance for results of the intercept at zero, which measures the degree of collinearity; data not shown). Third, because the models were developed to monitor T&O events for water quality management departments, we considered the costs and timeliness of monitoring and selected the models with parameters that could be easily collected based on sufficiently high R-Sq values.

The quality and fitness of the models for predicting the predominant T&O compounds were further evaluated using a residual scatter plot for each model. If the points were randomly dispersed around the zero line, a linear regression model was considered appropriate for the data; otherwise, a non-linear model was considered more appropriate [65].

Finally, it is necessary to test the applicability of the predictive models developed. However, in most field studies, complete data on the composition of the phytoplankton community, abiotic environmental factors and T&O compound concentrations are usually lacking. For these reasons, we used unpublished data collected across all of Taihu Lake in 2008 (Fig. 6) to test the applicability of the models developed here. We calculated the T&O values according to the models using the 2008 abiotic and biotic environmental data and compared these predicted T&O values with the T&O values observed in 2008.
