# Correction: Development of Models for Predicting the Predominant Taste and Odor Compounds in Taihu Lake, China

**DOI:** 10.1371/annotation/c86cbb15-dbb2-4fe8-b2fa-35604b6c4887

**Published:** 2013-03-19

**Authors:** Min Qi, Jun Chen, Xiaoxue Sun, Xuwei Deng, Yuan Niu, Ping Xie

There was an error in the Funding statement. 

The correct statement is: This study was jointly supported by the National Basic Research Program of China (973 Program) (2008CB418101) [http://www.973.gov.cn/AreaAppl.aspx], National Water Pollution Control and Management Technology Major Project (2012ZX07101-010) [http://nwpcp.mep.gov.cn/] and State Key Laboratory of Freshwater Ecology and Biotechnology (2011FBZ07) [http://eastlake.ihb.ac.cn/]. The funders had no role in study design, data collection and analysis, decision to publish, or preparation of the manuscript.

